# Longitudinal cognitive changes in patients with early Parkinson's disease and neuropsychiatric symptoms

**DOI:** 10.1111/cns.14173

**Published:** 2023-03-16

**Authors:** Detao Meng, Zhaohui Jin, Yixuan Wang, Boyan Fang

**Affiliations:** ^1^ Parkinson Medical Center, Beijing Rehabilitation Hospital Capital Medical University Beijing China

**Keywords:** cognitive impairment, neuropsychiatric symptoms, Parkinson's disease, prediction

## Abstract

**Aims:**

In this study, we aimed to investigate the effect of neuropsychiatric symptoms (NPS) on the rate of cognitive decline for both global cognition and specific cognitive domains in a cohort of patients from the Parkinson's Progression Markers Initiative (PPMI).

**Method:**

Prospectively longitudinal data were obtained from the PPMI cohort. NPS, including depression, anxiety, apathy, psychosis, impulse control disorders (ICDs), and cognition ability, were evaluated by a series of questionnaires. Linear mixed‐effects models were used to investigate the relationship between NPS and the rate of cognitive decline. Generalized estimating equations (GEEs) were used to investigate the relationship between NPS and the occurrence of mild cognitive impairment (MCI).

**Results:**

In total, 423 patients with Parkinson's disease (PD) were recruited at baseline and 395, 378, 366, 346, and 315 participants were followed up at 1, 2, 3, 4, and 5 years, respectively. Depression, anxiety, apathy, and psychosis were associated with global cognitive decline. Except for those with ICDs, patients with psychosis, depression, anxiety, and apathy were more likely to meet the criteria for MCI. Patients with depression and anxiety showed a progressive decline in four major cognitive domains. Apathy and ICDs were separately associated with a progressive decline in processing speed‐attention and memory, respectively.

**Conclusions:**

Neuropsychiatric symptoms, including psychosis, depression, anxiety, and apathy, could be used to predict future cognitive decline in patients with PD.

## Graphical abstract

This is the largest longitudinal study to study the relationship between cognitive impairment and neuropsychiatric symptoms (NPS). NPS, including psychosis, depression, anxiety, and apathy, could be used to predict future cognitive decline in patients with PD. There is no relationship between impulse control disorders and cognitive decline in early PD patients.

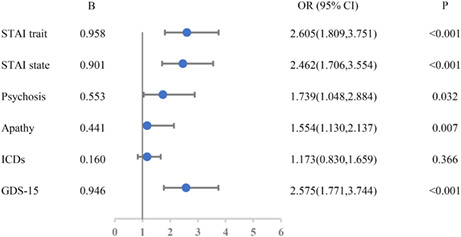

## 1 INTRODUCTION

Cognitive impairment (CI) is one of the most important non‐motor symptoms experienced by patients with Parkinson's disease (PD).^1^It can present before or at the time of a diagnosis of PD^2^, ^3^ and gradually worsens as the disease progresses.^4^ Even mild cognitive impairment (MCI) can impact a patient's activity of daily living and quality of life, thus causing a significant increase in the caregiver's burden.^5^

Although numerous studies have investigated non‐motor symptoms in PD patients, especially cognitive decline, little is known about CI in PD, particularly compared to motor symptoms. Our ability to predict cognitive decline in PD remains very limited. Considering the importance of cognitive dysfunction in PD patients, it is critical to identify factors that could predict a faster rate of global cognitive decline, such as neuropsychiatric symptoms (NPS). NPS included depression, anxiety, apathy, psychosis, and impulse control disorders (ICDs); these are regarded among the most common non‐motor symptoms in patients with PD.^6^ Some studies reported NPS negatively impact cognitive abilities in PD patients.^7^, ^8^, ^9^, ^10^, ^11^ However, in other studies, the relationship between NPS and cognitive function remained unclear.^3^, ^12^ Moreover, specific NPS may have differing effects on different cognitive domains.^9^, ^13^ In addition, most studies that investigated the relationship between cognitive dysfunction and NPS were cross‐sectional or case series studies that only included patients in the moderate to severe stages of disease.^13^ Furthermore, high‐quality longitudinal cohort studies are scarce and lack evidence from the early stages of disease. Therefore, in the present study, we analyzed the data from the Parkinson's Progression Marker's Initiative (PPMI), a longitudinal cohort that included 423 newly diagnosed PD patients who were followed up annually for up to 5 years. Our aim was to confirm the relationship between cognitive dysfunction and NPS.

Previous studies have used the PPMI database to investigate the incidence and progression of NPS and CI in PD patients. These studies reported that compared with healthy controls, patients with PD were more depressed, anxious, apathetic, and psychotic^3^ and that these symptoms increased significantly over time.^14^ A previous study showed that NPS increased by 6.2%–20.9% and that CI increased by 2.7%–6.2% by year 5 relative to baseline.^15^ No previous research has investigated the specific association between NPS and cognitive decline. Thus, the present study was designed to (i) confirm longitudinal relationships between NPS and cognitive decline in this longitudinal cohort and (ii) evaluate the influence of different NPS on cognitive domains over time.

## 2 METHODS

### 2.1 Study design

The data used in this study were acquired from the PPMI database (http://www.ppmi‐info.org).^16^ The PPMI is a multicenter, international, longitudinal cohort study that was conducted at 33 clinical sites, including the United States, Europe, Israel, and Australia. The PPMI study and protocols were approved by the local ethics committees and review boards at 33 clinical sites. Written informed consent was acquired from each participant before being included in the study. All patients were drug‐naïve at baseline and underwent clinical assessments. Our longitudinal study was conducted using PPMI data collected between January 2011 and December 2017; all patients were followed up for 5 years. This research was approved by the ethics committee of the Beijing Rehabilitation Hospital. All methods in this study were carried out in accordance with relevant guidelines and regulations.

### 2.2 Participants

A total of 423 PD patients were enrolled at baseline. At recruitment, participants were required to: (1) be over 30 years of age, (2) have dopamine transporter deficit on dopamine transporter imaging, (3) be competent to provide signed and informed consent, and (4) have been recently diagnosed with an asymmetric resting tremor or asymmetric bradykinesia or two of the following conditions: bradykinesia, resting tremor, and rigidity. All the PD patients recruited were drug‐naïve.

### 2.3 Motor assessment

Motor functions were assessed by the Movement Disorder Society‐Sponsored Revision of the Unified Parkinson's Disease Rating Scale (MDS‐UPDRS).^17^ The MDS‐UPDRS Part III is a disease‐relevant motor examination used to assess the motor experiences related to daily life.^17^ The clinical stage of PD was evaluated by the Hoehn & Yahr (H&Y) stage.^18^ According to Jankovic Subtype,^19^ the participants were divided into tremor dominant (TD), indeterminate, and postural instability/gait dominant (PIGD) types.

### 2.4 Cognitive assessments

Cognitive assessments included a global cognitive screening test with the Montreal Cognitive Assessment (MoCA)^20^ and a series of validated tests that examined four major areas of cognitive domains: memory, visuospatial functions, working memory‐executive functions, and attention‐processing speed. Memory was evaluated by the Hopkins Verbal Learning Test‐Revised (HVLT‐R),^21^ while visuospatial function was assessed by the Benton Judgment of Line Orientation (JOLO).^22^ Processing speed‐attention was evaluated by the Symbol‐Digit Modalities Test (SDMT) and working memory‐executive functions were evaluated by the Letter Number Sequencing and semantic fluency test.^23^ For all these tests, a higher score implied a better performance. The participants were divided into three groups (normal cognition, MCI, and dementia) according to the cognitive function test results. MCI was defined as having two or more HVLT total recall, HVLT recognition discrimination, JOLO, Letter Number Sequencing, semantic fluency test, or SDMT scores exceeding 1.5 standard deviations (SD) below normal, and without functional impairment due to CI.^24^ Dementia was defined as existing CI that could generate functional impairments that influenced the activity of daily living.^24^ All these cognitive assessments were estimated at baseline and follow‐up visits.

### 2.5 Neuropsychiatric symptoms

Depression was evaluated by the 15 items Geriatric Depression Scale (GDS‐15); a score ≥5 indicated clinically significant depression.^25^ Anxiety was evaluated by the State–Trait Anxiety Inventory (STAI); a score ≥40 on each subscale indicated clinically significant anxiety.^26^ ICDs were evaluated by the short version of the Questionnaire for Impulsive‐Compulsive Disorders in PD for ICDs and related behaviors (gambling, sexual, buying, eating, punding, hobbyism, walkabout, and the compulsive use of medication).^27^ Psychosis and apathy were separately assessed by the hallucinations/psychosis item and apathy item from the MDS‐UPDRS^17^ Part I. Any psychosis or apathy symptom graded 1 or more was considered as the presence of psychosis or apathy symptom.

### 2.6 Other assessments

Rapid eye movement sleep behavior disorder (RBD) was measured with the RBD Sleep Questionnaire^28^; a cutoff value of ≥6 was considered suggestive of REM sleep behavior disorder (RBD).

### 2.7 Statistical analysis

For the demographic and clinical characteristics at baseline, descriptive statistics were expressed as mean ± standard deviation (SD) for continuous variables and as percentage frequency for categorical variables. Data distribution and normality were evaluated with the Kolmogorov–Smirnov test in patients with and without MCI at baseline. Age, duration, age at onset, education, H&Y stage, and MDS‐UPDRS part III were all normally distributed and were compared by independent samples t‐tests in patients with and without MCI at baseline. Gender, race, motor subtype, STAI trait, STAI state, GDS‐15, ICDs, apathy, and psychosis was categorical variables and were analyzed by the chi‐squared test in patients with and without MCI at baseline.

Linear mixed‐effects models were used to investigate the relationship between baseline NPS and the rate of cognitive decline, including psychosis, depression, anxiety, apathy, and ICDs. These models interpreted the correlations between repeated measures and variables over time. The predictive power of NPS on cognitive decline was analyzed via interactions with visit time, thus revealing the influence of NPS on cognitive score changes over time. The following terms were used as independent variables in each model: the presence/absence of NPS at baseline, visit, and visit*NPS interaction term. The repeated cognitive test measures were used as the dependent variables. The presence/absence of NPS and covariates as fixed effects and the intercept as a random effect. Covariates included age, age at onset, sex, education, H&Y stage, duration, tremor/PIGD phenotype, MDS‐UPDRS III score, and RBD score.

Generalized estimating equations (GEEs) were used to investigate the relationship between baseline NPS and the occurrence of MCI. The GEE model could account for the reversion to normal that occurred in patients with MCI and the correlations among repeated measurements.^29^ The GEEs utilized the same covariates as described above.

Data were analyzed using SPSS version 21 (SPSS Inc.) and *p* < 0.05 was considered statistically significant.

## 3 RESULTS

In total, 423 PD patients were recruited at baseline and 395, 378, 366, 346, and 315 patients were followed up at 1, 2, 3, 4, and 5 years, respectively. The baseline demographic and clinical features of PD patients are shown in Appendix S1. The clinical characteristics were similar when compared between patients with and without CI, including age, race, duration, age at onset, education, H&Y stage, motor subtype, the severity of depression, QUIP, apathy, and psychosis. Patients with CI had a more severe UPDRS III score, and a higher proportion of females (*p* = 0.03), trait anxiety (*p* = 0.011), and state anxiety (*p* = 0.003) than those without CI.

**FIGURE 1:**
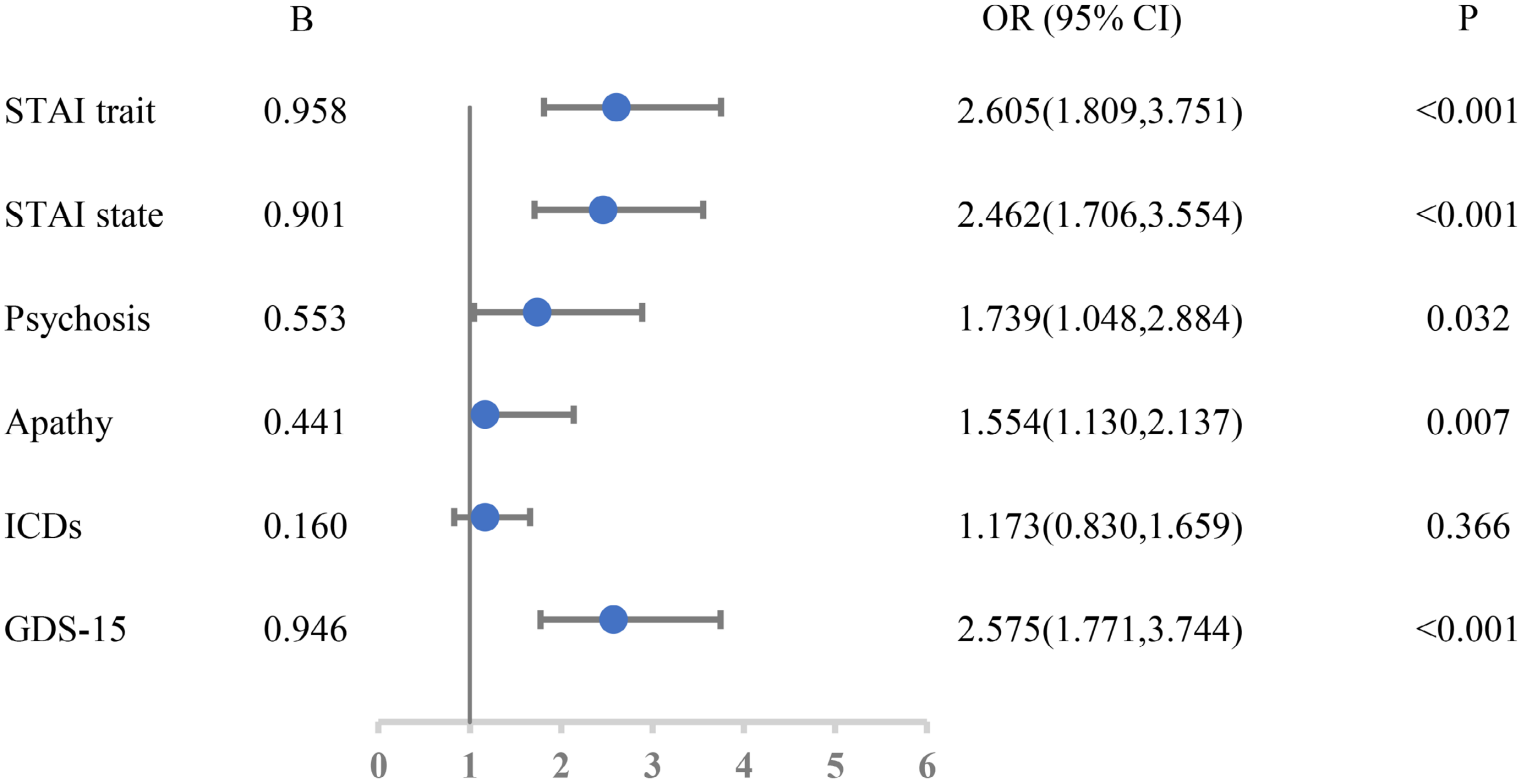
Forest plot showing the odds of MCI in patients with and without neuropsychiatric symptoms.

Cognitive decline was correlated with depression during follow‐up (Table 1). The MOCA score in depressed patients decreased by 0.638 points per annum when compared with non‐depressed patients (*p* < 0.001) (Table 1). With regard to specific cognitive tests, depressed patients experienced greater declines in semantic fluency, SDMT, HVLT‐R recognition discrimination, HVLT‐R delayed free recall, and JOLO (Table 1). There was no significant difference in Letter‐Number Sequencing between patients with and without depression (Table 1). The odds of MCI was significantly greater among depressed patients in comparison with those of non‐depressed patients (odds ratio [OR] = 2.575; 95% confidence interval [CI]: 1.771–3.744, *p* < 0.001) in the GEE model (Figure 1).

**TABLE 1:** The results of linear mixed‐effects models investigating the effect of depression on cognitive scores.

Dependent variable	*β*‐coefficient (95% CI)	*p*‐Value
MoCA	−0.638 (−0.968, −0.309)	<0.001
Semantic fluency	−1.757 (−2.868, −0.645)	0.002
SDMT	−2.451 (−3.495, −1.406)	<0.001
HVLT‐R recognition discrimination	−0.323 (−0.643, −0.004)	0.047
HVLT‐R delayed free recall	−0.749 (−1.078, −0.421)	<0.001
Letter‐Number Sequencing	−0.176 (−0.473, 0.121)	0.245
JOLO	−0.430 (−0.694, −0.166)	0.001

*Note*: In these models, GDS‐15 ≥5 is the independent variable, and the cognitive score is the dependent variable, Age, age at onset, sex, education, H&Y Stage, duration, tremor/PIGD phenotype, MDS‐UPDRS III score, and RBD score are covariates.

Abbreviations: GDS‐15, 15 item Geriatric Depression Scale; HVLT‐R, Hopkins Verbal Learning Test‐Revised; JOLO, Benton Judgment of Line Orientation; MoCA, Montreal Cognitive Assessment; SDMT, Symbol‐Digit Modalities Test.

Cognitive decline is also correlated with apathy over time (Table 2). PD patients with apathy experienced a reduction in MOCA score of 0.296 points per annum compared with those without apathy (*p* = 0.031) (Table 2). Patients with apathy experienced a significantly greater reduction in the SDMT (Table 2). There was no significant difference in semantic fluency, HVLT‐R recognition discrimination, HVLT‐R delayed free recall, Letter‐Number Sequencing, and JOLO between patients with and without apathy (Table 2). The odds of MCI were significantly greater in patients with apathy when compared with those without apathy (OR = 1.554; 95% CI: 1.130–2.137; *p* = 0.007) in the GEE model (Figure 1).

**TABLE 2:** The results of linear mixed‐effects models investigating the effect of apathy on cognitive scores.

Dependent variable	*β*‐coefficient (95% CI)	*p*‐Value
MoCA	−0.296 (−0.564, 0.028)	0.031
Semantic fluency	−0.511 (−1.408, 0.387)	0.264
SDMT	−0.994 (−1.834, −0.153)	0.021
HVLT‐R recognition discrimination	−0.127 (−0.388, −0.134)	0.388
HVLT‐R delayed free recall	−0.232 (−0.497, 0.034)	0.087
Letter‐Number Sequencing	−0.215 (−0.455, −0.024)	0.078
JOLO	−0.195 (−0.410, 0.019)	0.075

*Note*: In these models, any positive apathy score is the independent variable, and the cognitive score is the dependent variable. Age, age at onset, sex, education, H&Y Stage, duration, tremor/PIGD phenotype, MDS‐UPDRS III score, and RBD score are covariates.

Abbreviations: HVLT‐R, Hopkins Verbal Learning Test‐Revised; JOLO, Benton Judgment of Line Orientation; MoCA, Montreal Cognitive Assessment; SDMT, Symbol‐Digit Modalities Test.

Cognitive decline also correlated with psychosis over time (Table 3). Patients with psychosis experienced a reduction in the MOCA score of 0.708 points per annum compared with those without psychosis (*p* = 0.001). With regard to specific cognitive tests, there was no significant difference in semantic fluency, SDMT, HVLT‐R recognition discrimination, HVLT‐R delayed free recall, Letter‐Number Sequencing, or JOLO between patients with and without psychosis. The odds of MCI were significantly higher greater among patients with psychosis than those without psychosis (OR = 1.739; 95% CI: 1.048–2.884; *p* = 0.032) in the GEE model (Figure 1).

**TABLE 3:** The results of linear mixed‐effects models investigating the effect of psychosis on cognitive scores.

Dependent variable	*β*‐coefficient (95% CI)	*p*‐Value
MoCA	−0.708 (−1.137, −0.280)	0.001
Semantic fluency	−0.928 (−2.363, 0.507)	0.205
SDMT	−0.427 (−1.779, 0.925)	0.535
HVLT‐R recognition discrimination	−0.047 (−0.468, 0.373)	0.826
HVLT‐R delayed free recall	−0.308 (−0.733, 0.118)	0.156
Letter‐Number Sequencing	−0.011 (−0.395, 0.373)	0.956
JOLO	−0.219 (−0.562, 0.124)	0.211

*Note*: In these models, any positive psychosis score is the independent variable, and the cognitive score is the dependent variable. Age, age at onset, sex, education, H&Y Stage, duration, tremor/PIGD phenotype, MDS‐UPDRS III score, and RBD score are covariates.

Abbreviations: HVLT‐R, Hopkins Verbal Learning Test‐Revised; JOLO, Benton Judgment of Line Orientation; MoCA, Montreal Cognitive Assessment; SDMT, Symbol‐Digit Modalities Test.

Cognitive decline is also correlated with anxiety over time (Table 4). Patients with state anxiety experienced a reduction in MOCA score of 0.439 points per annum (*p* = 0.003) while patients with trait anxiety experienced a reduction in MOCA score of 0.346 points per annum (*p* = 0.029) when compared to those without state anxiety and trait anxiety. With regard to specific cognitive tests, patients with state anxiety experienced significantly greater declines in SDMT, HVLT‐R delayed free recall, Letter‐Number Sequencing, and JOLO (Table 4). Patients with trait anxiety experienced significantly greater declines in semantic fluency, SDMT, HVLT‐R delayed free recall, and JOLO (Table 4). The odds of MCI were significantly higher in patients with state anxiety when compared with those without state anxiety (OR = 2.462; 95% CI: 1.706–3.554; *p* < 0.001) and were significantly greater in patients with trait anxiety when compared with those without trait anxiety (OR = 2.605; 95% CI: 1.809–3.751; *p* < 0.001) in the GEE model (Figure 1).

**TABLE 4:** The results of linear mixed‐effects models investigating the effect of STAI state and STAI trait on cognitive scores.

Dependent variable	STAI state		STAI trait	
	*β*‐coefficient (95% CI)	*p*‐Value	*β*‐coefficient (95% CI)	*p*‐Value
MoCA	−0.439 (−0.734, −0.145)	0.003	−0.346 (−0.656, −0.036)	0.029
Semantic fluency	−0.913 (−1.906, 0.080)	0.072	−1.755 (−2.811, −0.698)	0.001
SDMT	−0.961 (−1.893, −0.029)	0.043	−2.394 (−3.374, −1.415)	<0.001
HVLT‐R recognition discrimination	−0.174 (−0.461, 0.113)	0.235	−0.127 (−0.424, 0.170)	0.401
HVLT‐R delayed free recall	−0.493 (−0.787, −0.199)	0.001	−0.530 (−0.839, −0.221)	0.001
Letter‐Number Sequencing	−0.278 (−0.542, −0.013)	0.040	−0.263 (−0.542, 0.016)	0.065
JOLO	−0.247 (−0.483, −0.010)	0.041	−0.529 (−0.778, −0.281)	<0.001

*Note*: In these models, STAI state ≥39 and STAI trait ≥39 is the independent variable and the cognitive score is the dependent variable. Age, age at onset, sex, education, H&Y Stage, duration, tremor/PIGD phenotype, MDS‐UPDRS III score, and RBD score are covariates.

Abbreviations: HVLT‐R, Hopkins Verbal Learning Test‐Revised; JOLO, Benton Judgment of Line Orientation; MoCA, Montreal Cognitive Assessment; SDMT, Symbol‐Digit Modalities Test; STAI, State–Trait Anxiety Inventory.

Cognitive decline was not correlated with QUIP over time in our model (Table 5). Only HVLT‐R recognition discrimination was associated with the cognitive decline and decreased by 0.280 points more per annum than those without QUIP (*p* = 0.043). There was no significant difference in MOCA, Semantic fluency, SDMT, HVLT‐R recognition discrimination, HVLT‐R delayed free recall, Letter‐Number Sequencing, and JOLO between patients with and without QUIP (Table 5). Finally, there is no significant difference in the odds of MCI with and without QUIP (OR = 1.173; 95% CI: 0.830–1.659; *p* = 0.366) in the GEE model (Figure 1).

**TABLE 5:** The results of linear mixed‐effects models investigating the effect of ICDs on cognitive scores.

Dependent variable	*β*‐coefficient (95% CI)	*p*‐Value
MoCA	0.017 (−0.262, −0.296)	0.905
Semantic fluency	0.192 (−0.736, 1.121)	0.685
SDMT	0.320 (−0.556, 1.196)	0.474
HVLT‐R recognition discrimination	−0.280 (−0.552, −0.009)	0.043
HVLT‐R delayed free recall	0.071 (−0.206, 0.349)	0.615
Letter‐Number Sequencing	−0.175 (−0.424, −0.074)	0.167
JOLO	0.091 (−0.132, 0.315)	0.423

*Note*: In these models, any positive score is the independent variable, and the cognitive score is the dependent variable. Age, age at onset, sex, education, H&Y Stage, duration, tremor/PIGD phenotype, MDS‐UPDRS III score, and RBD score are covariates.

Abbreviations: HVLT‐R, Hopkins Verbal Learning Test‐Revised; ICDs, impulse control disorders; JOLO, Benton Judgment of Line Orientation; MoCA, Montreal Cognitive Assessment; SDMT, Symbol‐Digit Modalities Test.

## 4 DISCUSSION

In this study of early PD patients untreated at baseline, we examined the longitudinal relationship between NPS and global cognition changes in a PPMI cohort, as well as the association between NPS and four major cognitive domains, including memory, visuospatial functions, working memory‐executive functions, and attention‐processing speed over time. We identified significant associations between NPS at baseline and the rate of cognitive decline over time (in years).

NPS are important clinical manifestation for PD patients.^30^ These symptoms are highly prevalent and can severely affect quality of life, for both the patients and their caregivers. Despite growing evidence suggesting a relationship between NPS and CI in PD patients, little is known of the specific aspects of this association.^9^ Depression, anxiety, and psychosis, including visual hallucinations, are widely accepted as independently risk factors for subsequent CI or dementia in PD patients.^9^, ^31^, ^32^ The independent influence of apathy on cognitive function has been investigated in previous studies^33^, ^34^; this research revealed that cognitive decline and/or dementia occurred more frequently in apathetic patients.

ICDs are common neuropsychiatric complications experienced by PD patients.^35^ Related pathological behaviors, such as gambling, compulsive eating, shopping, or uninhibited sexual behavior, can have adverse effects on a patient's quality of life. However, the specific relationship between ICDs and cognitive decline has yet to be fully investigated. Some studies found that patients with ICDs experience a faster rate of cognitive decline,^36^, ^37^ whereas other studies did not identify this association.^38^ In addition, Siri et al. reported that patients with ICDs were not associated with CI at baseline but manifested with a relatively lower degree of cognitive decline in long‐term cognitive follow‐up.^39^ Our current findings agreed with that of Bentivoglio^38^ in that we found that ICDs were not associated with global cognitive decline and that patients with ICDs were not more likely to develop MCI in the PPMI cohort. Although our study sheds some light on this issue, there is a clear need for further investigation.

In our study, we also investigated the influence of different NPS on cognitive domains over time. We found that patients with depression and anxiety had a lower score in attention‐processing speed and memory, along with visuospatial and executive tests. Apathy was found to be associated with a progressive decline in processing speed‐attention. QUIP also demonstrated a link with memory impairment, but with a large *p*‐value (*p* = 0.043); as such, this relationship should be interpreted cautiously. Psychosis was not associated with cognitive domain changes in this longitudinal cohort. In previous work, Pirogovsky‐Turk et al.^9^ and Gryc et al.^40^ also investigated the potential for this relationship in a longitudinal cohort. Pirogovsky‐Turk et al. found that anxiety and depression were the strongest predictors of longitudinal decline with regard to measures of verbal and visual learning; there was no significant association with a decline in other cognitive domains.^9^ Gryc et al. found that psychosis was significantly associated with a diagnosis of PD dementia and with a reduced time to dementia longitudinally.^40^ The differences between these earlier results and our current findings could be explained by different methodologies and study populations.^13^ Because few longitudinal cohort studies have investigated these relationship, our current findings need to be verified further in other cohorts.

A key question to consider is why patients with NPS are more likely to experience cognitive decline; the mechanisms underlying this association remain unclear. One possible reason might be due to PD‐related disruption in frontostriatal circuits.^9^, ^41^ This phenomenon might be due to the common neuroanatomical substrates associated with NPS and CI.^42^, ^43^, ^44^, ^45^, ^46^ A significant relationship has been detected between an elevated total neuropsychiatric inventory score and the frontal atrophy level in PD patients.^43^, ^44^, ^46^ Furthermore, frontal cortical thinning was reported to represent a useful marker for the conversion of early dementia^45^, ^47^; abnormalities in the frontostriatal pathway have also been reported to contribute to CI in PD patients.^48^ Common changes in the frontal cortex may explain the greater susceptibility of PD patients with NPS to cognitive decline. Another possible reason may be due to dysregulation of the hypothalamic–pituitary–adrenal (HPA)‐axis in PD patients.^9^, ^49^, ^50^, ^51^ The HPA‐axis is a key neuroendocrine signaling system involved in physiological homeostasis and stress response.^49^ Disturbances of this system lead to chronically elevated levels of cortisol; this can subsequently damage neurons in the hippocampus, an anatomical region that is critical for cognitive function.^9^ Therefore, patients are prone to develop cognitive dysfunction after presenting with NPS.

Some limitations of this study must be acknowledged. First, both NPS and CI can be influenced by dopamine replacement therapy and the use of antipsychotic medications. Although the participants were drug‐naïve PD patients at baseline, we still cannot neglect the potential influence of drugs in this study. Second, as this was a longitudinal cohort study that featured 5 years of follow‐up, some questionnaires and rating scales were missing due to the long follow‐up time. Finally, multiple NPS frequently co‐occur in PD patients, especially depression and anxiety; it is difficult to differentiate between these multiple manifestations.^13^, ^52^ However, despite these limitations, our research findings are still of great significance and may help to identify PD patients for special trial interventions to prevent cognitive decline.

In conclusion, our study confirmed that NPS, including depression, anxiety, and apathy and psychosis exhibit longitudinal relationships with CI in PD patients. These patients experienced greater rates of cognitive decline over time. The specific relationships between ICDs and CI warrant further investigation. Our study also demonstrated an association between NPS (especially depression and anxiety) and several impaired cognitive domains. Although dysfunction of the front striatal circuits and HPA‐axis could explain this phenomenon, the underlying mechanisms warrant further study.

## AUTHOR CONTRIBUTIONS

Study design: BYF; MDT; data acquisition: MDT; data analysis: MDT; statistical analysis: MDT; manuscript editing: MDT, BYF, JZH, and WYX; manuscript review: BYF, JZH, and WYX. All authors approved the final version of the study.

## FUNDING INFORMATION

This work was supported by grants from the National Natural Science Foundation of China (82004431) and the Science and Technology Development Fund of Beijing Rehabilitation Hospital, Capital Medical University (2021‐005). The funding body had no role in protocol design, statistical analysis, and manuscript preparation.

## CONFLICT OF INTEREST STATEMENT

The authors declare that the research was conducted in the absence of any commercial or financial relationships that could be construed as a potential conflict of interest.

## Supporting information


Appendix S1

## Data Availability

The data that support the findings of this study are available from the corresponding author upon reasonable request.
