# Supplementary material for: Longitudinal cognitive changes in patients with early Parkinson's disease and neuropsychiatric symptoms
Source: CNS Neurosci Ther. 2023 Mar 16;29(8):2259–66. doi: 10.1111/cns.14173 (PMC10352866; doi:10.1111/cns.14173)
Supplement: Supplementary file 1 — Appendix S1 [file CNS-29-2259-s001.docx]

**Supplementary Material**

**Appendix1.** Demographic and clinical features of PD patients with and without MCI

|  | Entire cohort (n=422) | No MCI (n=356） | MCI (n=66) | P-value |
| --- | --- | --- | --- | --- |
| Age | 61.66 ± 9.71 | 61.5 ± 9.9 | 62.8 ± 8.4 | 0.291^a^ |
| Gender |  |  |  | 0.030^b^ |
| Man | 277 (65.48%) | 226 (63.5%) | 51 (77.3%) |  |
| Female | 146 (34.52%) | 130 (36.5%) | 15 (22.7%) |  |
| Race |  |  |  | 0.129^b^ |
| White | 391 (92.43%) | 332 (93.26%) | 58 (87.88%) |  |
| Non-white | 32 (7.57%) | 24(6.74%) | 8(12.12%) |  |
| Duration | 6.65 ± 6.50 | 6.5 ± 6.4 | 7.7 ± 7.2 | 0.162^a^ |
| Age onset | 59.65 ± 9.96 | 59.4 ± 10.2 | 61.0 ± 8.7 | 0.237^a^ |
| Education | 15.56 ± 2.97 | 15.7 ± 2.8 | 15.0 ± 3.7 | 0.122^a^ |
| H&Y stage | 1.57 ± 0.51 | 1.56 ± 0.51 | 1.64 ± 0.48 | 0.237^a^ |
| MDS-UPDRS part III | 20.89 ± 8.85 | 20.4 ± 8.7 | 23.7 ± 9.0 | 0.004^a^ |
| Motor subtype, n (%) | |  |  | 0.492^b^ |
| TD | 299 (70.85%) | 248 (69.7%) | 50 (76.9%) |  |
| PIGD | 76 (18.01%) | 67 (18.8%) | 9 (13.8%) |  |
| Indeterminate | 47 (11.14%) | 41 (11.5%) | 6 (9.2%) |  |
| STAI trait |  |  |  | 0.011^b^ |
| <39 | 330 (78.20%) | 286 (80.34%) | 43 (66.15%) |  |
| >=39 | 92 (21.80%) | 70 (19.66%) | 22 (33.85%) |  |
| STAI state | |  |  | 0.003^b^ |
| <39 | 311 (73.70%) | 272 (76.4%) | 38 (58.5%) |  |
| >=39 | 111 (26.30%) | 84 (23.6%) | 27 (41.5%) |  |
| GDS-15 |  |  |  | 0.145^b^ |
| Not depressed (<5) | 364 (86.05%) | 310 (87.1%) | 53 (80.3%) |  |
| Depressed (≥5) | 59 (13.95%) | 46 (12.9%) | 13 (19.7%) |  |
| ICDs | |  |  | 0.064^b^ |
| Negative | 335 (79.38%) | 288 (80.9%) | 46 (70.8%) |  |
| Any 1 or more disorders | 87 (20.62%) | 68 (19.1%) | 19 (29.2%) |  |
| Apathy |  |  |  | 0.692^b^ |
| Negative | 352 (83.22%) | 295 (82.9%) | 56 (84.8%) |  |
| Any positive score | 71 (16.78%) | 61 (17.1%) | 10 (15.2%) |  |
| Hallucinations and psychosis | |  |  | 0.453^b^ |
| Negative | 410 (96.93%) | 346 (97.2%) | 63 (95.5%) |  |
| Any positive score | 13 (3.07%) | 10 (2.8%) | 3 (4.5%) |  |

Note: Data are presented as the mean±SD except categorical variables. Categorical variables were reported as n (%). a: independent-samples t-test; b: χ2 test.

H&Y stage, Hoehn and Yahr stage; MDS-UPDRS, Movement Disorder Society-Sponsored Revision of the Unified Parkinson’s Disease Rating Scale; TD, tremor dominant; PIGD, postural instability/gait dominant; STAI, State-Trait Anxiety Inventory; GDS-15, 15 item Geriatric Depression Scale; ICDs, impulse control disorders.
